# A Comparative Transcriptomic with UPLC-Q-Exactive MS Reveals Differences in Gene Expression and Components of Iridoid Biosynthesis in Various Parts of *Gentiana macrophylla*

**DOI:** 10.3390/genes13122372

**Published:** 2022-12-15

**Authors:** Yuhang Kou, Xiaoying Yi, Zhuo Li, Yun Ai, Siting Ma, Qianliang Chen

**Affiliations:** 1 College of Life Sciences, Northwest University, Xi’an 710069, China; 2 Xi’an Institute for Food and Drug Control, Xi’an 710054, China; 3 Biomedicine Key Laboratory of Shaanxi Province, Northwest University, Xi’an 710069, China

**Keywords:** *Gentiana macrophylla*, transcriptome, iridoid synthesis pathway, functional genes, UPLC-Q-Exactive MS

## Abstract

*Gentiana macrophylla* Pall. (*G. macrophylla*)—a member of the family Gentianaceae—is a well-known traditional Chinese medical herb. Iridoids are the main active components of *G. macrophylla*, which has a wide range of pharmacological activities such as dispelling wind, eliminating dampness, clearing heat and asthenic fever, hepatoprotective and choleretic actions, and other medicinal effects. In this study, a total of 67,048 unigenes were obtained by transcriptomic sequencing analysis of *G. macrophylla.* A BLAST analysis showed that 48.21%, 33.66%, 46.32%, and 32.62% of unigenes were identified in the NR, Swiss-Prot, eggNOG, and KEGG databases, respectively. Twenty-five key enzymes were identified in the iridoid biosynthesis pathway. Most of the upregulated unigenes were enriched in flowers and leaves. The trustworthiness of the transcriptomic data was validated by real-time quantitative PCR (qRT-PCR). A total of 22 chemical constituents were identified by ultra-high performance liquid chromatography-quadrupole-electrostatic field Orbitrap mass spectrometry (UPLC-Q-Exactive MS), including 10 iridoids. A correlation analysis showed that the expression of *7-DLH* and *SLS* was closely related to iridoids. The expression of *7-DLH* and *SLS* was higher in flowers, indicating that flowers are important for iridoid biosynthesis in *G. macrophylla*.

## 1. Introduction

*Gentiana macrophylla* Pall. is a perennial herb of the genus Gentiana, mainly distributed in Shaanxi, Gansu, and Tibet, in China [1]. In traditional Chinese medicine, the roots of *G. macrophylla* are used, while their flowers are also used in Tibetan medicine [2]. Research shows that iridoids are the main active ingredients of *G. macrophylla* [3]. It has many medical effects, such as dispelling wind, eliminating dampness, clearing heat and asthenic fever, hepatoprotective and choleretic actions, etc. [4]. Owing to its high medical value, its uncontrolled exploitation leads to wild resources being extremely scarce, and it is listed by the Chinese government as third-class protected wild herb [5]. The studies on *G. macrophylla* are mainly focused on its active constituents, pharmacological effects, germplasm resources, and pharmacognosy. The active components in medicinal plants are formed by their unique biosynthetic pathways. The biosynthesis process of iridoids can be divided into three stages: the synthesis of intermediates (i.e., IPP and DMAPP), terpenoid synthesis (i.e., catalyzing various intermediates or terpenoids from IPP and DMAPP), and final modification (i.e., the complex structural modification of iridoids end products) [6]. Based on previous research [7] on iridoid biosynthesis in *G. macrophylla*, we used transcriptomic and metabolomic analyses to further reveal the iridoid biosynthesis in different parts of *G. macrophylla*.

Transcriptomic sequencing (RNA-Seq) refers to the sequencing and analysis of all or part of the RNA in cells or tissues. For RNA transcripts, it reflects the expression of all genes in different life stages, different physiological states, and different environmental conditions of an organism [8]. At present, high-throughput transcriptomic sequencing has been widely used in the exploration of secondary metabolic pathways and the identification of functional genes in medical plants such as *Gynostemma pentaphyllum* [9], *Eucommia ulmoides* [10], *Cornus officinalis* [11], and *Cupressus gigantea* [12]. Metabolomics is a fast-growing technology that has effectively contributed to many plant-related sciences and drug discovery [13]. The metabolites of medicinal plants are numerous and complex, and they can be adequately separated and identified by metabolomics technology. Thus, multi-omics are more applied to reveal the mechanism of biosynthesis in plants, seeking the relationship between genes and metabolites; for instance, key genes of flavonoid synthesis were higher expressed in processed leaf tissues than in non-processed leaves, leading to a high content of flavonoids in *Agave lechuguilla* waste biomass [14]. Furthermore, in medicinal plants, it was found that the two cultivars of *Dendrobium officinale* with differences in volatile terpenoid products were caused by the differential expression of terpene synthases [15].

In this study, the Illumina NovaSeq™ 6000 sequencing platform was used to sequence and then analyze the transcriptomes of the roots, stems, leaves, and flowers of *G. macrophylla.* We identified the components in different parts of *G. macrophylla* via UPLC-Q-Exactive mass spectrometry. This study further illustrates the different iridoid contents in *G. macrophylla* based on the expression of genes, providing a theoretical basis for its iridoid biosynthesis pathways.

## 2. Materials and Methods

### 2.1. Plant Materials

*G. macrophylla* plants were collected in the town of Badu, Long County, Baoji, Shaanxi Province, in July 2021 (latitude: 34°71′4444″ N; longitude: 106°82′6931″ E). Samples were identified by Professor Shuonan Wei of Northwestern University as *Gentiana macrophylla* Pall. Three plants were taken as biological replicates for analysis. Each sample was divided into roots, stems, leaves, and flowers. Then, the samples were split into two parts. One part was used for UPLC-Q-TOF MS analysis and was quickly dried in an oven at 100 °C after being shredded [16]. The other part was collected in RNase-free tubes and quick-frozen in liquid nitrogen, before being stored at −80 °C in a refrigerator for later use. Specimens and reserved samples were kept in the Shaanxi Provincial Key Laboratory of Biomedicine.

### 2.2. RNA Extraction and Sequencing

Total RNA was extracted using TRIzol reagent (Invitrogen, CA, USA) and an RNA purification kit, following the manufacturers’ procedures. The total RNA quantity and purity were analyzed using Bioanalyzer 2100 and RNA 1000 Nano LabChip Kit (Agilent, CA, USA), with a RIN number >7.0. The cleaved RNA fragments were reverse-transcribed to create the final cDNA library in accordance with the protocol for the mRNA-Seq sample preparation kit (Illumina, San Diego, CA, USA); the average insert size for the paired-end libraries was 300 bp (±50 bp). Then, we performed the paired-end sequencing on an Illumina NovaSeq™ 6000 at LC Sciences, Houston, TX, USA, following the vendor’s recommended protocol.

### 2.3. Transcript Assembly and Unigene Functional Annotation

Firstly, in-house Cutadapt [17] and Perl scripts were used to remove low-quality and undetermined bases. Then, sequence quality was verified using FastQC (http://www.bioinformatics.babraham.ac.uk/projects/fastqc/, accessed on 1 October 2021). De novo assembly of the transcriptome was performed using Trinity 2.4.0 [18]. Trinity grouped transcripts into clusters based on shared sequence content. The longest transcript in the transcript cluster was selected as the ‘gene’ sequence (also known as unigene).

All of the assembled unigenes were aligned against the non-redundant protein database (NR) (https://www.ncbi.nlm.nih.gov/, accessed on 2 October 2021), Gene Ontology (GO) (http://geneontology.org/, accessed on 3 October 2021), Swiss-Prot (https://www.expasy.org/swiss-prot, accessed on 4 October 2021), Kyoto Encyclopedia of Genes and Genomes (KEGG) (www.genome.jp/kegg, accessed on 5 October 2021), and eggNOG (http://eggnog5.embl.de, accessed on 6 October 2021) databases using DIAMOND [19], with a threshold E-value <0.00001.

### 2.4. Analysis of Differentially Expressed Genes

Salmon [20] was used to determine expression levels for unigenes by calculating TPM [21]. The differentially expressed unigenes were selected with log2 (fold change) > 1 or log2 (fold change) < −1 based on statistical significance (*p*-value < 0.05) using the R package edgeR [22]. The identified differentially expressed genes were subjected to the GO and KEGG pathway enrichment analyses to further analyze the main differential functions between sites.

### 2.5. Identification of Transcription Factors

According to the priority order of the NR, Swiss-Prot, KOG, and KEGG, unigenes were aligned with the above protein library (E value < 1 × 10^−5^) using BLASTx [23]. ESTScan [24] was used to predict the coding regions. The predicted unigenes’ encoded protein sequences were compared with the plant transcription factor database (Plant-TFDB) using hmmscan to search for transcription factor families and their members.

### 2.6. qRT-PCR Validation of Key Genes in the Biosynthesis of Iridoid Compounds

qRT-PCR was performed using the QuantGene 9600 System (Bioer Technology, ZJ, CHN) and the SYBR^®^ Green Premix Pro Taq HS qPCR Kit (Accurate Biology). Special primers were designed for different genes using Primer Premier 5.0 (Table S1). The RNAs from the four parts (i.e., roots, stems, leaves, and flowers) were extracted and reverse-transcribed into cDNA using the Evo M-MLV RT Mix Kit with gDNA Clean for qPCR (Accurate Biology). The polymerase chain reaction conditions were as follows: 95 °C for 30 s, 40 cycles of 95 °C for 5 s, and 60 °C for 30 min. All qRT-PCR analyses were repeated in three biological and three technical replicates. The *UBC 13* gene was used as a reference. The relative expression levels of the selected genes were determined using the 2^−ΔΔCt^ method [25].

### 2.7. Analysis of Constituents by UPLC-Q-Exactive MS

#### 2.7.1. Sample Preparation

Samples of four parts (roots, stems, leaves, and flowers) were dried and ground. The powder (sieved using a No.3 sifter) was weighed to precisely 0.25 g, placed in a 10 mL volumetric flask, and methanol was added to the scale line, before it was sonicated (power 500 W, frequency 40 kHz) for 30 min and cooled to room temperature, after which its capacity was fixed with methanol and it was filtered. The filtrate was filtered through a 0.22 μm microporous membrane.

#### 2.7.2. UPLC-Q-Exactive MS Analysis

The chromatographic conditions were as described in the study [26], with some modifications for optimization. The chromatographic column was an OSAKA SODA CAPCELL PAK MG C18 column (150 mm × 2.0 mm, 5 μm), the mobile phase was acetonitrile (A), 0.1% formic acid water (B), and the elution gradient was as follows: 0~2 min, 5% A; 2~20 min, 5~15% A; 20~38 min, 15~40% A; 38~50 min, 40~80% A; and 50~56 min, 80~5% A. The volume flow rate was 0.3 mL/min, the column temperature was 35 °C, and the injection volume was 10 μL.

The ion source was a HESI source with the following parameters: positive and negative ion detection modes, a sheath gas flow rate of 4.58 L/min, an auxiliary gas flow rate of 7.97 L/min, a spray voltage of 3.44 KV, a capillary temperature of 320 °C, an ion transport tube temperature of 350 °C, and an auxiliary gas temperature of 350 °C. The scan modes were as follows: full MS/dd-MS2, full MS resolution 70,000, dd-MS2 resolution 17,500, and a scan range m/z 100–800. The collision gas was nitrogen (purity > 99.99%) and the collision energy was 30 eV.

#### 2.7.3. UPLC-Q-Exactive MS Data Acquisition and Analysis

The mass spectrometry data were analyzed using Xcalibur software (Thermo Fisher Scientific, Waltham, MA, USA) to derive the possible molecular formulae from the high-resolution mass spectral information, with mass spectral deviations in the range of δ < 4 × 10^−6^. The parent ion was determined from a relevant literature search combined with the ion abundance > 1 × 10^6^ in the full-scan spectrum in positive and negative ion modes. Identification of the products was based on retention times, parent ions, and secondary ion fragments, and it was confirmed by a literature search.

### 2.8. Correlation Analysis between Expression of Key Enzyme Genes and Constituents

Six key enzyme genes (*HDR*, *GPPS*, *G8O*, *SLS*, *7-DLGT*, and *7-DLH*) were selected through iridoid biosynthesis pathways. Abbreviations of the enzymes are listed in Table S2. The enzyme genes’ qRT-PCR data were processed via the 2^−ΔΔCT^ method, with the roots treated as a control group. The data were correlated with the peak areas of the 14 iridoids of *G. macrophylla*. A clustering correlation heatmap with signs was constructed using the OmicStudio tools (https://www.omicstudio.cn, accessed on 7 July 2022)

### 2.9. Statistical Analysis

Graphing with GraphPad Prism 8.0 (GraphPad Software Inc., San Diego, CA, USA), results were presented as means ± standard error of the means (S.E.M.). Statistical analyses were performed by one-way ANOVA followed by Dunnett’s post-hoc test. A *p*-value < 0.05 was considered statistically significant.

## 3. Results

### 3.1. RNA-Seq and De Novo Transcriptome Assembly

A total of 79.89 GB of sequence data were generated, including 20.50 GB from the roots, 21.35 GB from the stems, 18.64 GB from the leaves, and 19.40 GB from the flowers (Table S3). A principal component analysis (PCA) showed that the values of PC1 and PC2 were 48.00 and 25.85%, respectively, and the root group was clearly separated from the other groups (Figure S1).

**Figure 1:**
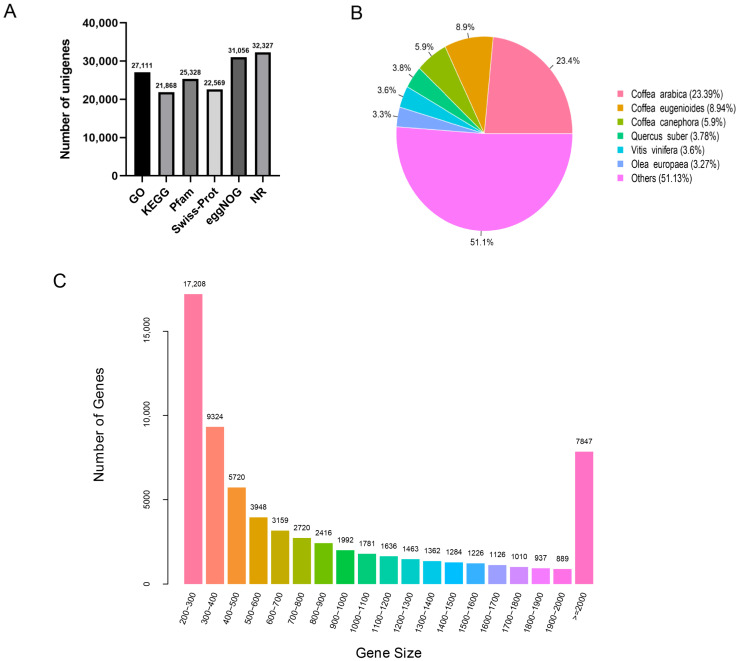
Illumina sequencing and transcriptomes of different tissues in *G. macrophylla*: (**A**) The annotation of unigenes based on various databases. (**B**) The species distribution of the annotated unigenes. (**C**) The detailed information of the assembled unigenes.

After assembling the valid reads, a total of 67,048 unigenes were obtained, with sizes ranging from 200 to 15,651 bp and an average size of 917 bp (Figure 1C). There were 46,487 transcripts (69.34%) in the size range of 200–1000 bp, 12,714 (18.96%) at 100–2000 bp, and 7847 (11.70%) > 2000 bp. The resulting unigenes were sorted by length from high to low, and the length (N50) at half of the total length was 1571 bp.

### 3.2. Functional Annotation of Unigenes

The 67,048 assembled unigenes were aligned using BLASTx in the six databases of NR, GO, KEGG, Pfam, Swiss-Prot, and eggNOG. The annotation results are summarized in Figure 1A.

In the comparison with the NR database, 32,327 unigenes were annotated, accounting for 48.21% of the total. It can be seen that *G. macrophylla* has the highest homology with *Coffea arabica*, followed by *Coffea eugenioides*, *Coffea canephora*, *Vitis vinifera*, and *Olea europaea* (Figure 1B).

**Figure 2:**
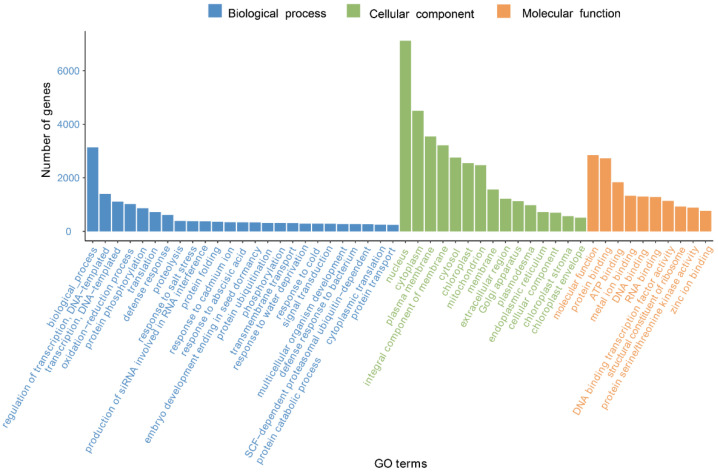
GO classification of *G. macrophylla* transcriptomic unigenes.

In the GO database, there are three systematically defined ways of describing the functions of gene products, namely the molecular function, biological process, and cellular component. As shown in Figure 2, a total of 27,111 unigenes were classified by GO annotation and divided into fifty functional groups in three categories. Among all categories, the nucleus category in the category of cellular components was the most annotated, accounting for 29.6% of the total annotations, followed by the cytoplasm (16.3%), and biological process categories (13.1%).

**Figure 3:**
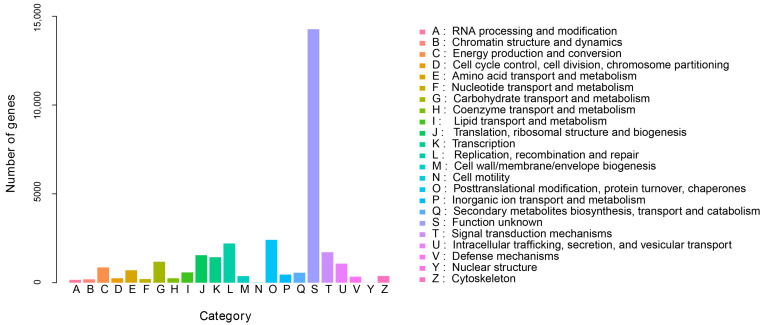
The eggNOG annotation of *G. macrophylla* transcriptomic unigenes.

**Figure 4:**
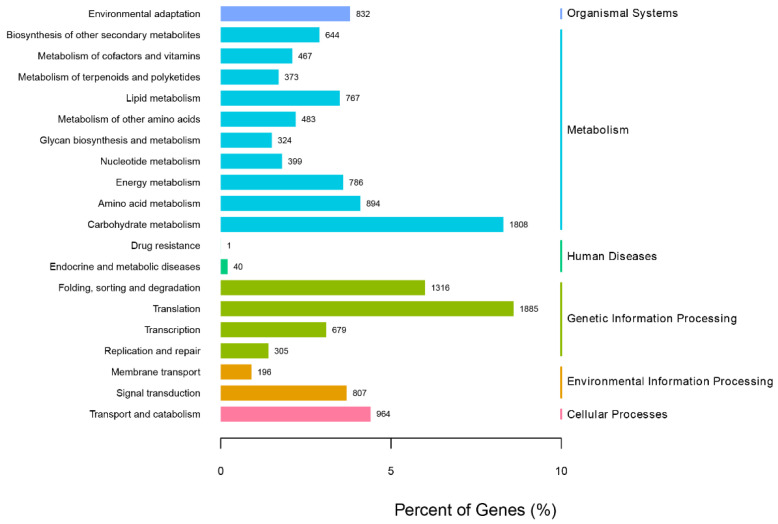
The KEGG pathway analysis of *G. macrophylla* transcriptomic unigenes.

In order to further analyze the function of unigenes in the transcriptome of *G. macrophylla*, the eggNOG functional classification analysis was performed. As shown in Figure 3, a total of 23 different eggNOG functional groups were obtained, including most life activities. The number of genes predicted by general functions was the largest, with 3389 unigenes; 17,167 unigenes were annotated in the KEGG database, involving a total of six major branches of the KEGG metabolic pathway (Figure 4). The top three annotated subcategories were translation, carbohydrate metabolism, and folding sorting and degradation, accounting for 7.82%, 7.68%, and 5.94% of the total annotations in the database, respectively.

### 3.3. Correlation Analysis of Secondary Metabolism

Most medical components are from secondary metabolites; therefore, secondary metabolism is closely related to medicinal value. In the *G. macrophylla* transcriptome, a total of 2170 unigenes were involved in the 128 standard KEGG secondary metabolism pathways, of which 92 unigenes were involved in the terpenoid backbone biosynthesis (Table 1).

**Figure 5:**
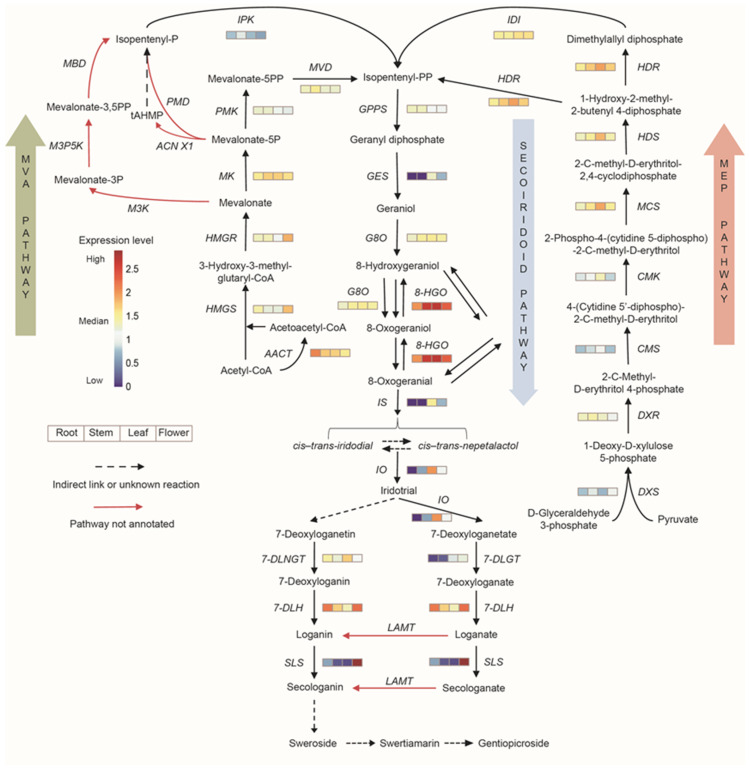
Expression analysis of genes involved in iridoid biosynthesis. Different color blocks represent the normalized gene expression levels (log10 (TPM+1)) in different tissues of *G. macrophylla*. The blocks from left to right represent roots, stems, leaves, and flowers, respectively. Red: higher expression; blue: lower expression.

As special terpenoids, iridoids also have similar synthetic pathways and are synthesized from geranyl pyrophosphate via complex ring opening, rearrangement, cyclization, and glycosylation processes. In total, 102 unigenes were annotated to 25 enzymes involved in iridoid synthesis pathways. The expression of enzyme genes in the biosynthetic pathways of iridoids is shown in Figure 5. Most of the genes showed higher expression in leaves and flowers.

### 3.4. Differential Gene Analysis

**Figure 6:**
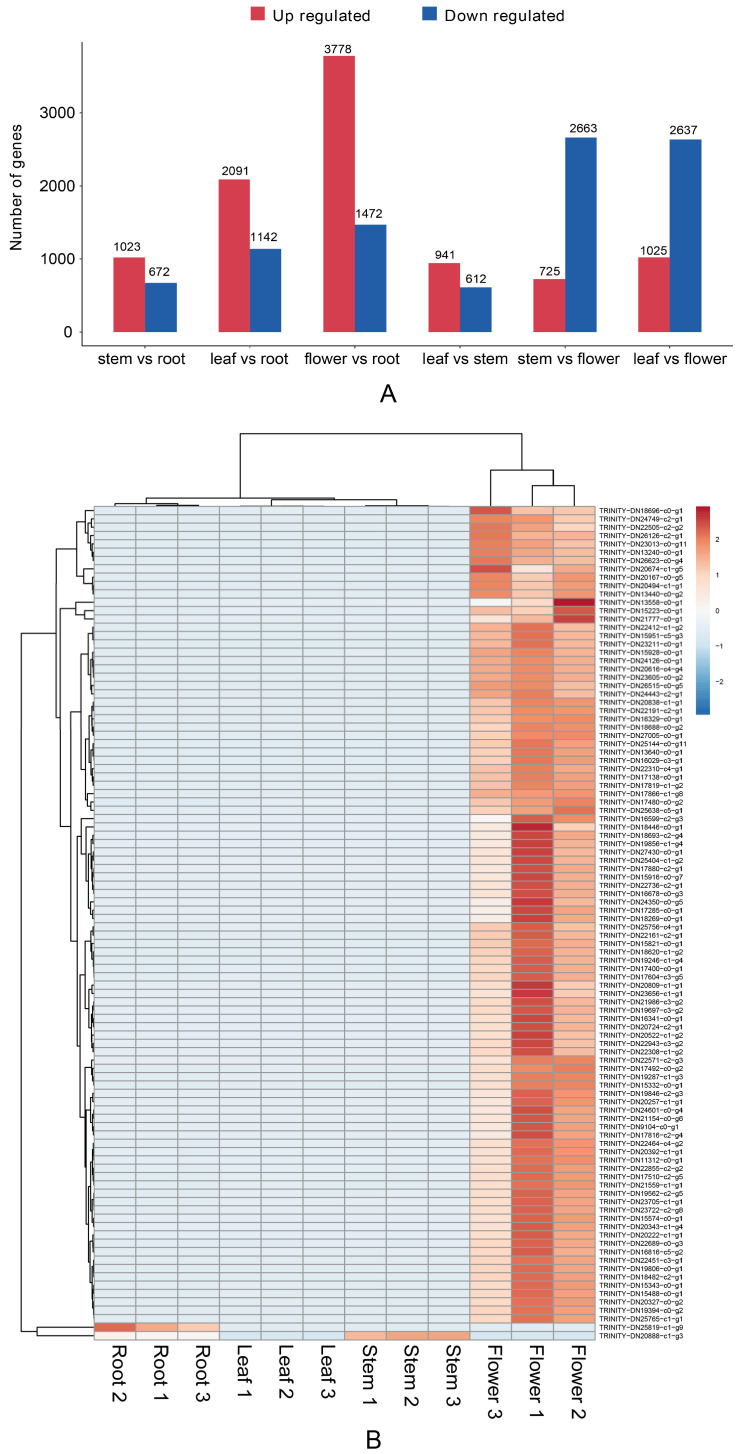
Expression of differential unigenes in *G. macrophylla*: (**A**) A summary upregulated and downregulated unigenes between sets of two specified samples. (**B**) Heatmap of differentially expressed genes from different tissues. Each column in the figure represents one sample, while each row represents one gene. The colors indicate the normalized gene expression levels (log10 (TPM+1)) in different tissues. Red and blue represent high and low expression levels, respectively.

Differential gene analysis was performed on the transcriptomic data of the roots, stems, leaves, and flowers (Figure 6A). In the pairwise comparisons between different parts, significant differences in transcription were observed. Compared with the above-ground parts, most of the differential genes in the roots were downregulated. A total of 5244 differential genes were identified between the roots and flowers, including 1466 upregulated genes and 3778 downregulated genes. According to the cluster analysis of differential genes (Figure 6B), the expression of genes in flowers was much higher than in other parts. This shows that the most vigorous parts of the physiological activity are flowers.

### 3.5. Analysis of Transcription Factors

**Figure 7:**
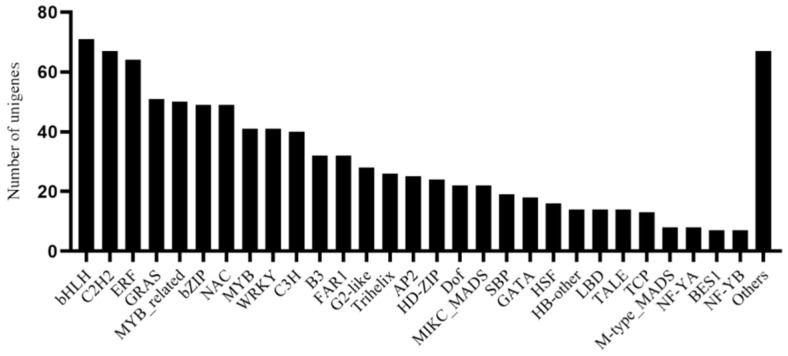
Classification of transcription factor families by analysis of the *G. macrophylla* transcriptome.

The transcription factor (TF) analysis of all unigenes in the transcriptome of *G. macrophylla* predicted that there were 940 unigenes belonging to 55 families. The most frequent TF type was bHLH, accounting for 7.55%, followed by C2H2, accounting for 7.13%, and ERF, accounting for 6.81% (Figure 7).

### 3.6. Validation of Key Enzyme Genes Using qRT-PCR

**Figure 8:**
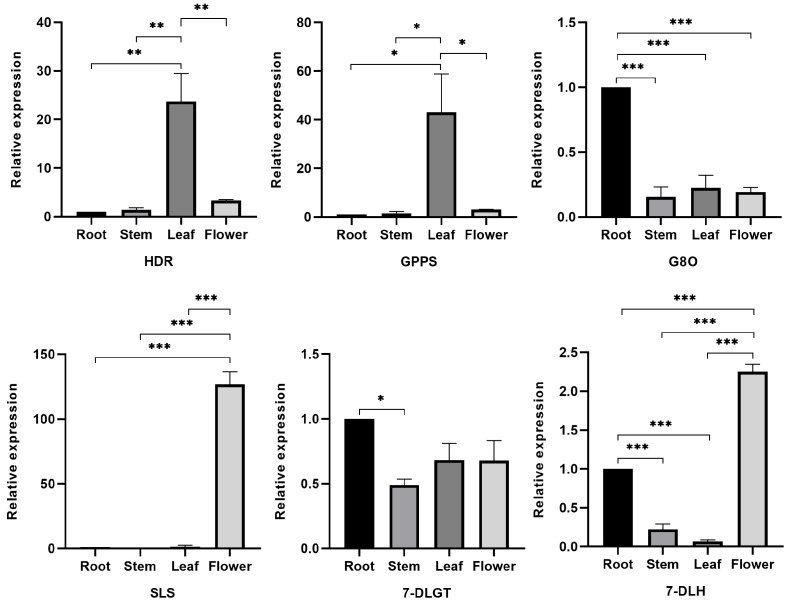
qRT-PCR analysis of six key enzyme genes (*HDR*, *GPPS*, *G8O*, *SLS*, *7-DLGT*, and *7-DLH*) which were involved in iridoid biosynthesis. The relative expressions of the genes were normalized against the *UBC 13* gene as an internal control; roots were set as the reference. Significance codes: * *p* < 0.05, ** *p* < 0.01, and *** *p* < 0.001. Data are represented as means ± S.E.M of *n* = 3 replicates.

To validate the transcriptomic analysis data and provide a better understanding of the biosynthesis of iridoids in *G. macrophylla*, we selected six key enzymes in the iridoids pathway to examine their different expressions in four parts of *G. macrophylla* by using qRT-PCR (Figure 8). The relative expression of the *HDR* and *GPPS* genes was the highest in leaves and the lowest in roots. The *7-DLGT* and *G8O* genes had the highest relative expression in roots and the lowest in stems. The *7-DLH* and *SLS* genes had the highest relative expression in flowers and the lowest in leaves. The expression trends were consistent with the results of the transcriptomic analysis (Figure S2). It was confirmed that transcriptomic analysis can accurately reflect the physiological situation of *G. macrophylla*.

### 3.7. Metabolite Analysis of G. macrophylla by UPLC-Q-Exactive MS

**Figure 9:**
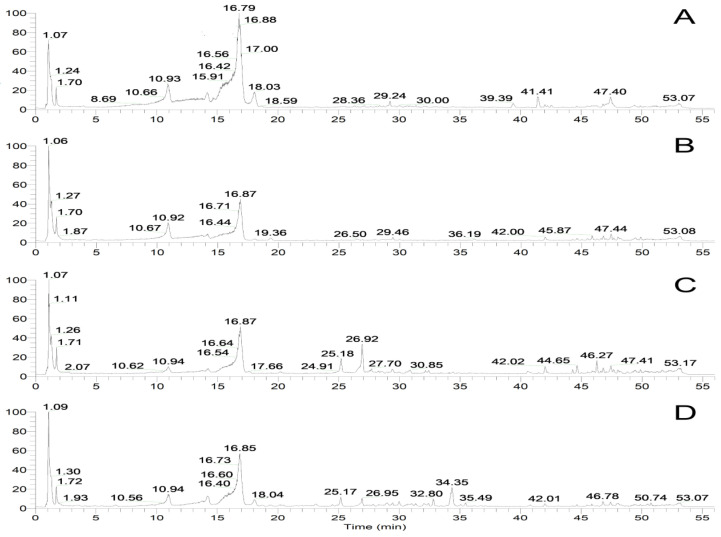
MS TIC chromatograms of roots (**A**), stems (**B**), leaves (**C**), and flowers (**D**) of *G macrophylla* determined by UPLC-Q-Exactive MS in negative ion mode.

The total ion flow diagram of the mass spectrometric base peaks determined via (-) ESI-MS is shown in Figure 9. A total of twenty-two compounds were identified from the mapping of each part, as shown in Table 2; ten of them were iridoids, four were flavonoids, two were triterpenes, two were phenylpropanoids, and four were others. 

**Figure 10:**
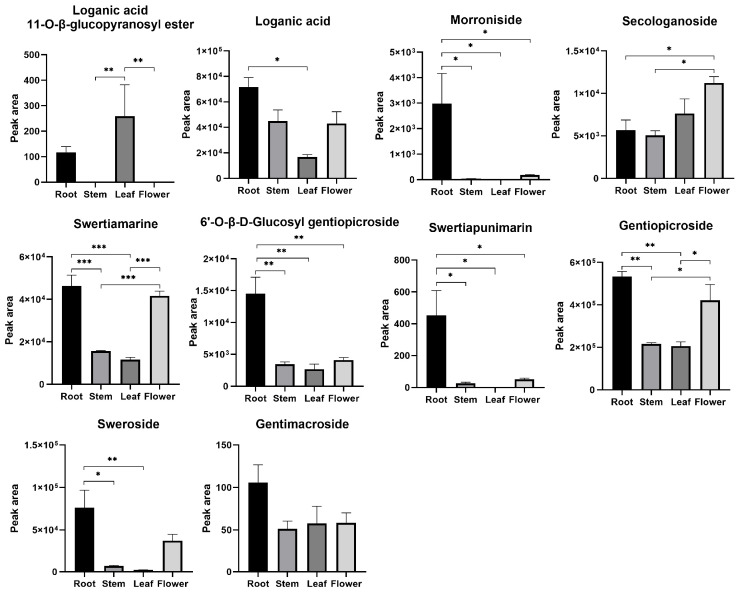
Relative contents of iridoids in different parts of *G. macrophylla* by the peak area of chromatograms. * *p* < 0.05, ** *p* < 0.01, and *** *p* < 0.001. Data are represented as means ± S.E.M of *n* = 3 replicates.

A loganic acid 11-O-β-glucopyranosyl ester was not identified in stems or flowers. Morroniside and swertiapunimarin were not detected in the leaves. Most of the iridoids were abundant in roots (Figure 10).

### 3.8. Correlation Analysis between the Expression of Key Enzyme Genes and Contents of Iridoids

**Figure 11:**
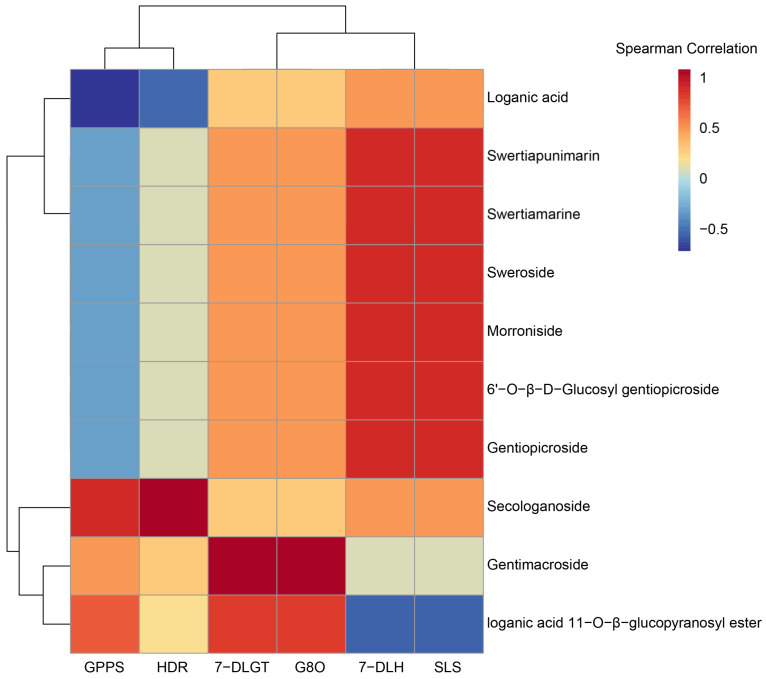
Cluster analysis of the expression of six key enzyme genes (*HDR*, *GPPS*, *G8O*, *SLS*, *7-DLGT*, and *7-DLH*) related to the contents of ten iridoids. Red and blue represent positive and negative correlations, respectively.

A correlation analysis was performed on the expression of six enzyme genes and the contents of ten iridoids in different parts of *G. macrophylla* (Figure 11). The results showed that *GPPS* and *HDR* were clustered and highly correlated with the secologanoside content. The expression of *7-DLH* and *SLS* was significantly correlated withmost iridoids. Iridoids *7-DLGT* and *G8O* were more strongly correlated with the content of the loganic acid 11-O-β-glucopyranosyl ester and Gentimacroside.

## 4. Discussion

Iridoids are the main bioactive products of *G. macrophylla* and have important medicinal value [37]; hence, their biosynthetic pathways merit further clarification—especially the relationship between enzyme genes’ expression and the contents of iridoids in different parts of *G. macrophylla*. Therefore, transcriptomic and metabolomic experiments were conducted to further explore the relationships and differences in iridoids between parts of *G. macrophylla*. A total of 67,048 unigenes were identified using the Illumina NovaSeq™ 6000 platform. Compared with a previous study [7], more genetic information on *G. macrophylla* was obtained. The GO catalogs and proportions of annotated unigenes were similar to related species such as *G. lhassica* [38], *G. waltonii*, and *G. robusta* [39]. The annotation of 136 standard KEGG metabolic pathways and 55 transcription families suggested that *G. macrophylla* involves a very complex transcriptional regulatory mechanism.

Through screening of rate-limiting enzymes in iridoid biosynthetic pathways by their differential expression in *G. macrophylla*, six key enzyme genes were selected that were involved in the upstream, midstream, and downstream of the pathway to verify the transcriptome data via qRT-PCR. The results confirmed that the transcriptomic analysis was reliable.

All too often, the investigation of gene expression remains the major trend in unraveling the regulatory mechanisms of metabolic pathways [40]. From annotation information, we identified 25 enzyme genes involved in iridoid biosynthesis, finding that most of them showed higher expression in leaves and flowers. In the formation of IPP intermediates, the MEP pathway dominates in *G. macrophylla*. These findings are consistent with those of a recently published work [41]. *HDR* is the last key enzyme on the MEP pathway, playing an important regulatory role in terpenoid synthesis [42]. The high expression of *HDR* in leaves suggests that IPP may be mainly synthesized in leaves. This is consistent with *Oncidium orchid* [43] and *Arabidopsis* [44]. The clustering analysis showed that there were far more differentially expressed genes in flowers than in other parts. As most of the enzyme genes of iridoids were highly expressed in leaves and flowers, we speculate that the iridoid components of *G. macrophylla* are mainly synthesized in the aboveground parts and then transported to roots for storage.

UPLC-Q-Exactive mass spectrometry was used not only to analyze the iridoids among different parts of *G. macrophylla*, but also to identify their components. Based on the results of the relative contents of iridoids in different parts of *G. macrophylla*, the iridoids were more abundant in the roots than in other parts. This is consistent with the results of *Gentiana crasicaulis* [36]. Loganic acid and gentiopicroside are the content detection items named in the 2020 edition of the *Chinese Pharmacopoeia* [45], representing the main active components of *G. macrophylla*; we found that their contents showed no significant differences between flowers and roots. This is reasonable based on the use offlowers in Tibetan and Mongolian medicine.

The results of the correlation analysis on the expression of key enzyme genes and iridoid contents showed gentiopicroside was the most important representative iridoid of *G. macrophylla*, and its content was inseparable from the expression of *7-DLH* and *SLS*. This provides a feasible idea of increasing the expression levels of *7-DLH* and *SLS* enzyme genes, which may lead to higher contents of gentiopicroside. In addition, the expression of *7-DLH* and *SLS* was extremely high in flowers compared to other parts. This indicates that the flowers are important for iridoid biosynthesis in *G. macrophylla*.

There are many studies showed that the secondary metabolism of plants is closely related to the environment [46,47]. Shaanxi Province, as the genuine producing area of *G. macrophylla*, has unique geographical and climatic conditions that have positive contributions to the accumulation of iridoids. However, its mechanism still remains to be explored. Additionally, gene expression control is critical to increase the production of enzymes, fine-tune metabolic pathways, and reliably express synthetic pathways [48]. In the future, we hope to verify the effects of differentially expressed genes on iridoid biosynthesis by controlling them, as well as to further investigate the mechanisms of iridoid transport in *G. macrophylla*.

## 5. Conclusions

In this study, a comparative transcriptomic with UPLC-Q-Exactive MS revealed differences in the gene expression and components of iridoid biosynthesis in various parts of *G. macrophylla*. According to the GO and KEGG databases, the 25 enzyme genes were identified in the iridoid biosynthesis pathway, and their differential expression resulted in the differential content of the 10 iridoids in various parts of *G. macrophylla*. Iridoids *7-DLH* and *SLS* showed a highly positive correlation with most other iridoids. These findings provide a comprehensive genetic resource that can enable improvements in our understanding of the regulation of iridoids’ biosynthesis and accumulation at the molecular level.

## Figures and Tables

**Table 1:** The KEGG pathway annotation analysis of *G. macrophylla* transcriptomic unigenes associated with secondary metabolism.

No.	Pathway	Number of Unigenes	Proportion %	KEGG ID
1	Phenylpropanoid biosynthesis	354	16.31	K09753
2	Flavonoid biosynthesis	129	5.94	K00660
3	Ubiquinone and other terpenoid-quinone biosyntheses	96	4.42	K12501
4	Terpenoid backbone biosynthesis	92	4.24	K05906
5	Phenylalanine, tyrosine, and tryptophan biosynthesis	78	3.59	K00500
6	Tropane, piperidine, and pyridine alkaloid biosynthesis	65	3.00	K08081
7	Fatty acid biosynthesis	64	2.95	K00665
8	Stilbenoid, diarylheptanoid, and gingerol biosynthesis	63	2.90	K13065
9	Sesquiterpenoid and triterpenoid biosynthesis	62	2.86	K15803
10	Cutin, suberine, and wax biosynthesis	56	2.58	K15403
11	Isoquinoline alkaloid biosynthesis	56	2.58	K14454
12	Biosynthesis of unsaturated fatty acids	50	2.30	K00507
13	Diterpenoid biosynthesis	48	2.21	K04125
14	Valine, leucine, and isoleucine biosynthesis	33	1.52	K00826
15	Indole alkaloid biosynthesis	26	1.20	K21026
16	Zeatin biosynthesis	21	0.97	K00279
17	Monoterpenoid biosynthesis	21	0.97	K15095
18	Monobactam biosynthesis	18	0.83	K12524
19	Flavone and flavonol biosynthesis	17	0.78	K05280
20	Benzoxazinoid biosynthesis	16	0.74	K13229
21	Betalain biosynthesis	15	0.69	K15777
22	Anthocyanin biosynthesis	12	0.55	K21383
23	Phenazine biosynthesis	7	0.32	K01657

**Table 2:** Compounds identified in *G. macrophylla* by UPLC-Q-Exactive MS.

No.	Ion Mode	t_R_/min		Excimer Ion Peaks		Molecular Formula	Adduct Ions	Ppm	Maior Fragment Ions (m/z)	Compounds	Parts
		Sample	Control	Measured (m/z)	Calculated (m/z)						
1	−	1.10		341.1094	341.1089	C_12_H_22_O_11_	[M−H]^−^	1.38	178.0560, 161.0448, 131.0339, 119.0335, 101.0229, 89.0228, 71.0122	Sucrose [27]	R, S, L, F
2	−	1.68		191.0191	191.0197	C_6_H_8_O_7_	[M−H]^−^	−3.30	159.3513, 134.9712, 111.0074, 87.0072, 67.0173, 59.0122, 57.0330	Citric Acid [27]	R, S, L, F
3	−	6.95		583.1870	583.1880	C_22_H_34_O_15_	[M+HCOO]^−^	−1.66	375.1304, 313.1309, 213.0761, 169.0865, 113.0229, 59.0122	Loganic acid 11-O-β-glucopyranosyl ester [4]	R, L
4	−	10.98	10.90	375.1299	375.1297	C_16_H_24_O_10_	[M−H]^−^	0.61	213.0764, 169.0861, 151.0754, 133.0647, 113.0231, 95.0487, 69.0329	Loganic acid [28]	R, S, L, F
5	+	11.69		429.1366	429.1367	C_17_H_26_O_11_	[M−Na]^−^	−0.23	267.0845, 235.0575, 203.0521, 185.0418, 110.6607, 79.2134	Morroniside [29]	R, S, F
6	−	13.78		389.1104	389.1089	C_16_H_22_O_11_	[M−H]^−^	3.78	319.0093, 2199.2400, 183.0659, 165.0549, 121.0645, 69.0329, 59.0122	Secologanoside [30]	R, S, L, F
7	−	14.21	14.16	419.1201	419.1195	C_16_H_22_O_10_	[M+HCOO]^−^	1.43	302.6173, 179.0552, 149.0597, 141.0181, 119.0336, 113.0230, 89.0228, 59.0122	Swertiamarine [31]	R, S, L, F
8	−	14.71	14.60	563.1631	563.1618	C_22_H_30_O_14_	[M+HCOO]^−^	2.38	221.0665, 193.0498, 179.0552, 161.0445, 131.0337, 101.0229, 89.0228	6’-O-β-D-Glucosyl gentiopicroside [31]	R, S, L, F
9	+	15.66		521.1862	521.1865	C_22_H_32_O_14_	[M+H]^+^	−0.58	251.4391, 197.0808, 179.0703, 151.0751, 127.0389, 111.0801	Swertiapunimarin [32]	R, S, F
10	−	16.79	16.83	401.1093	401.1089	C_16_H_20_O_9_	[M+HCOO]^−^	0.92	324.6015, 219.0556, 149.0597, 121.0648, 113.0231, 93.0333, 89.0228	Gentiopicroside [31]	R, S, L, F
11	+	16.81		177.0545	177.0546	C_10_H_8_O_3_	[M+H]^+^	−0.68	147.0440, 131.0491, 121.0647, 119.0497, 103.0543, 91.0492, 79.0544	Erythrocentaurine [33]	R, S, L, F
12	+	16.81		195.0649	195.0652	C_10_H_10_O_4_	[M+H]^+^	−1.49	177.0549, 149.0598, 131.0490, 121.0648, 103.0542, 91.0543, 79.0543	Ferulic acid [34]	R, S, L, F
13	−	18.10	18.23	403.1254	403.1246	C_16_H_22_O_9_	[M+HCOO]^−^	2.03	312.8494, 205.4160, 151.0749, 125.0229, 89.0229, 81.0329	Sweroside [31]	R, S, L, F
14	−	20.10		593.1524	593.1512	C_27_H_30_O_15_	[M−H]^−^	2.04	557.1314, 473.1095, 431.1017, 341.0662, 311.0567, 282.0520	Saponarin [35]	R, S, L, F
15	−	26.94		431.0991	431.0984	C_21_H_20_O_10_	[M−H]^−^	1.69	341.0669, 311.0566, 283.0620, 269.0458, 239.0713, 163.0392, 117.0332	Isovitexin [35]	R, S, L, F
16	+	29.26		235.0961	235.0965	C_13_H_14_O_4_	[M+H]^+^	−1.66	217.0858, 189.0907, 174.0675, 159.0804, 145.0647, 129.0698, 91.0542	2-methoxyanofinic acid [34]	R, S, L, F
17	−	29.27		603.1943	603.1931	C_25_H_34_O_14_	[M+HCOO]^−^	2.06	323.0986, 263.0774, 189.0912, 161.0961, 119.0336, 101.0229, 89.0228	Macrophylloside D [31]	R, S, L, F
18	−	33.09		301.0357	301.0354	C_15_H_10_O_7_	[M−H]^−^	1.06	193.0136, 151.0024, 149.0233, 107.0124, 83.0121	Quercetin [30]	R, S, L, F
19	−	35.35		285.0407	285.0405	C_15_H_10_O_6_	[M−H]^−^	0.84	257.0463, 192.0056, 159.0079, 151.0025, 108.0200, 83.0122	Kaempferol [30]	R, S, L, F
20	−	36.72		531.1522	531.1508	C_26_H_28_O_12_	[M−H]^−^	2.64	315.0727, 297.0625, 189.0910, 153.0182, 109.0280	Gentimacroside[3]	R, S, L, F
21	−	48.00		471.3488	471.3480	C_30_H_48_O_4_	[M−H]^−^	1.74	471.3486, 439.8552, 218.8751, 101.9868, 79.8463	Corosolic acid [36]	R, S, L, F
22	−	51.76		455.3539	455.3531	C_30_H_48_O_3_	[M−H]^−^	1.82	455.3540, 229.2049, 177.5234, 151.2601, 141.7751	Oleanic acid [36]	R, S, L, F

## Data Availability

The RNA-seq data have been submitted to the BIG Data Center of the Chinese Academy of Sciences (http://bigd.big.ac.cn, accessed on 1 October 2021) with accession number CRA007607.

## Supplementary Materials

The following supporting information can be downloaded at https://www.mdpi.com/article/10.3390/genes13122372/s1, Table S1: Primer names and sequences were used in this study; Table S2: Abbreviations of pathways and enzymes; Table S3: Transcriptome sequencing results of *G. macrophylla*; Figure S1: The principal components analysis (PCA) of four parts of *G. macrophylla*; Figure S2: The expression of six key enzyme genes (*HDR*, *GPPS*, *G8O*, *SLS*, *7-DLGT*, and *7-DLH*) by transcriptome analysis of *G. macrophylla*.
