# Supplementary material for: A Comparative Transcriptomic with UPLC-Q-Exactive MS Reveals Differences in Gene Expression and Components of Iridoid Biosynthesis in Various Parts of Gentiana macrophylla
Source: Genes (Basel). 2022 Dec 15;13(12):2372. doi: 10.3390/genes13122372 (PMC9778098; doi:10.3390/genes13122372)
Supplement: Supplementary file 1 [file genes-13-02372-s001.zip › Supplementary/Supplementary Figuers.pptx]

## Slide 1
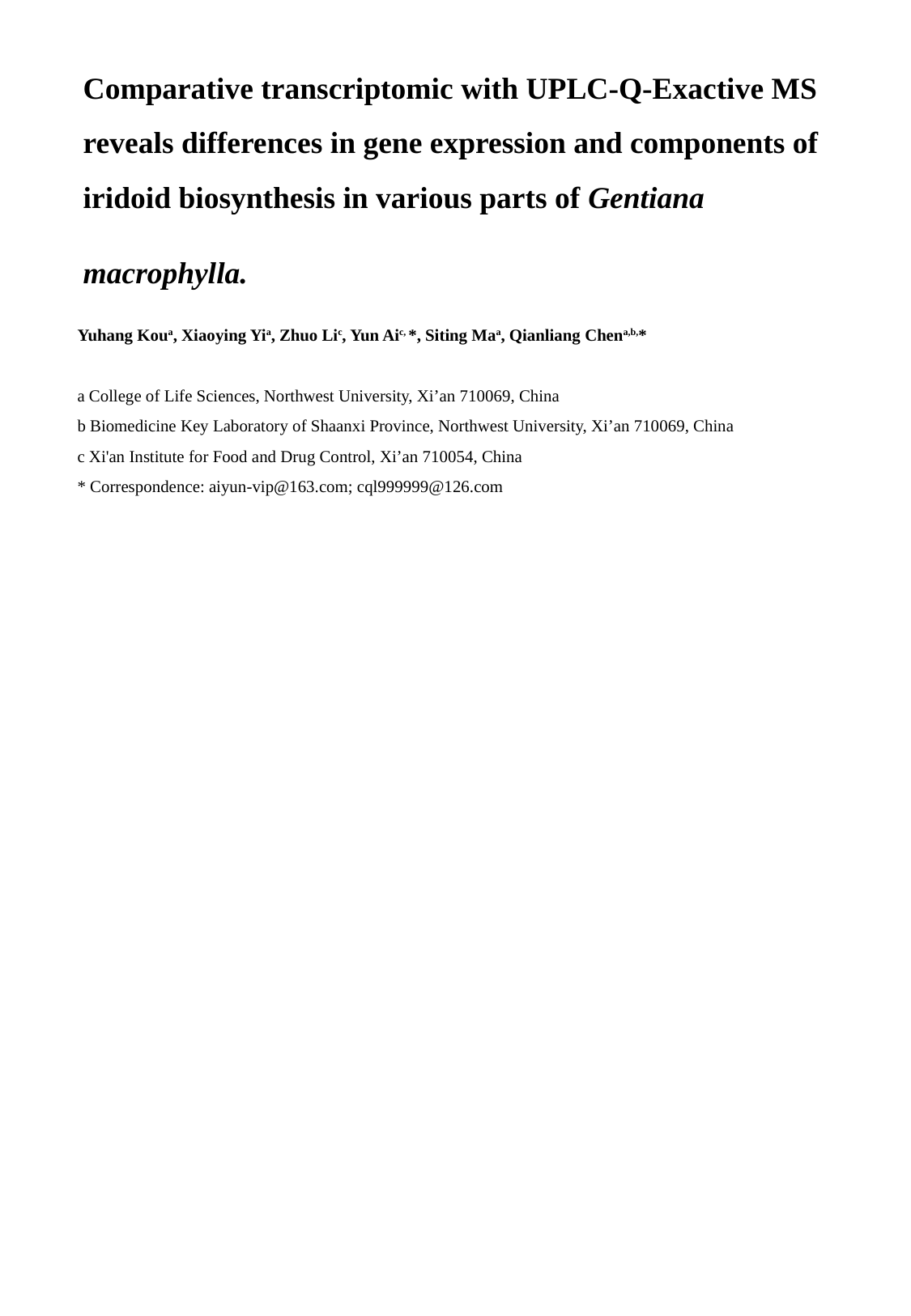

Comparative transcriptomic with UPLC-Q-Exactive MS reveals differences in gene expression and components of iridoid biosynthesis in various parts of Gentiana
macrophylla.
Yuhang Koua, Xiaoying Yia, Zhuo Lic, Yun Aic, *, Siting Maa, Qianliang Chena,b,*
a College of Life Sciences, Northwest University, Xi’an 710069, China
b Biomedicine Key Laboratory of Shaanxi Province, Northwest University, Xi’an 710069, China
c Xi'an Institute for Food and Drug Control, Xi’an 710054, China
* Correspondence: aiyun-vip@163.com; cql999999@126.com

## Slide 2
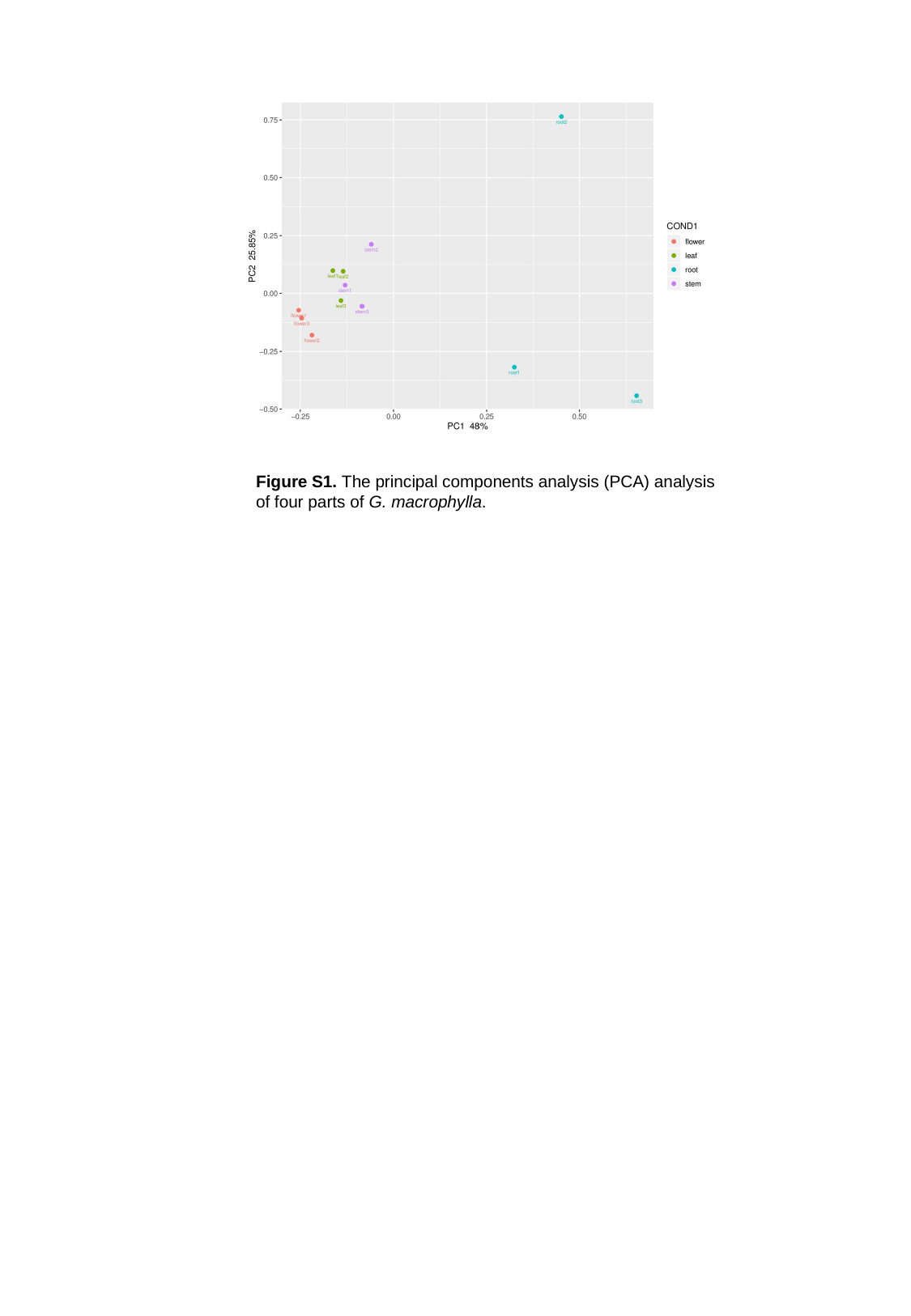

Figure S1. The principal components analysis (PCA) analysis of four parts of G. macrophylla.

## Slide 3
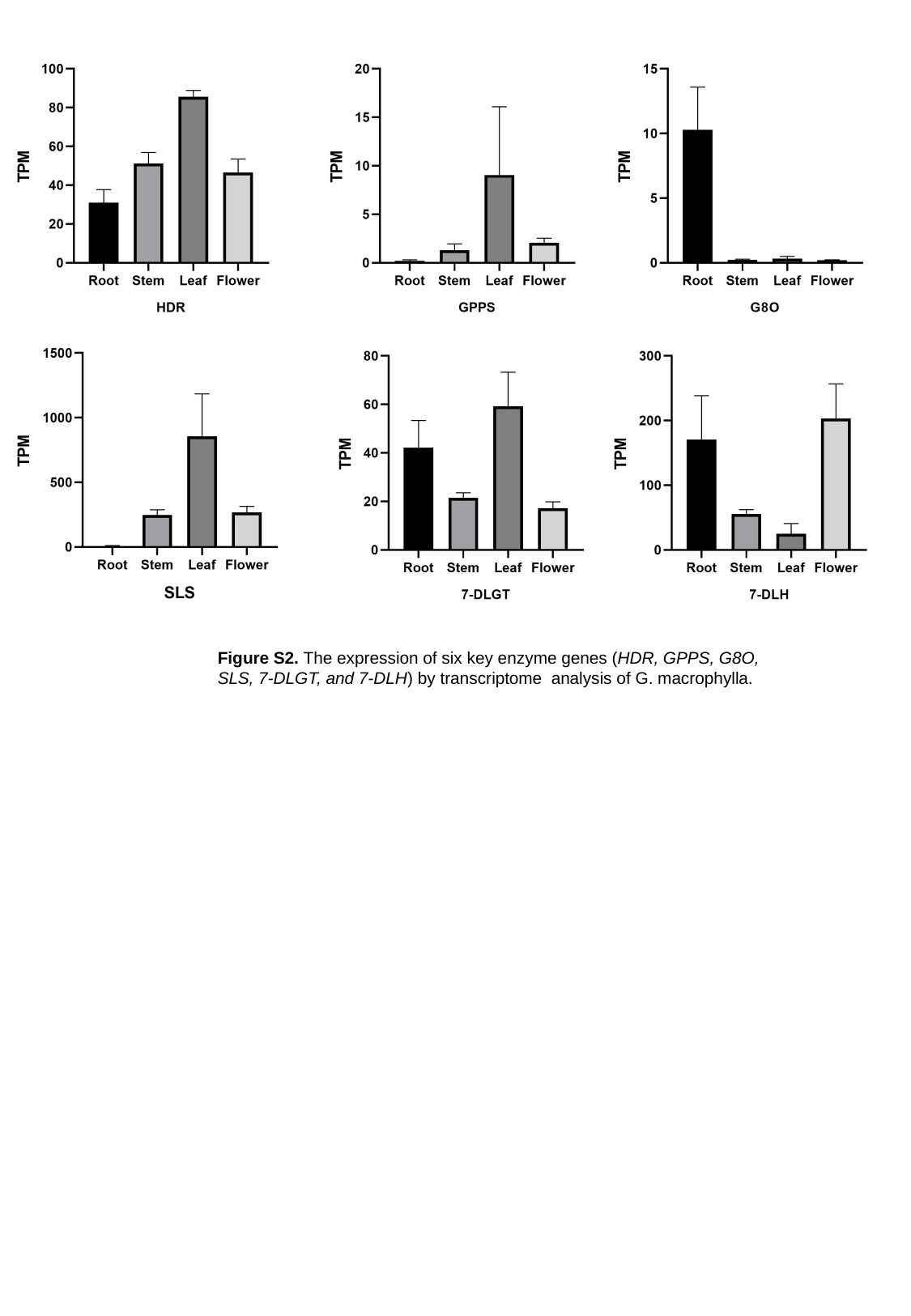

Figure S2. The expression of six key enzyme genes (HDR, GPPS, G8O, SLS, 7-DLGT, and 7-DLH) by transcriptome analysis of G. macrophylla.
